# Anti-Prion Activity of a Panel of Aromatic Chemical Compounds: *In Vitro* and *In Silico* Approaches

**DOI:** 10.1371/journal.pone.0084531

**Published:** 2014-01-06

**Authors:** Natalia C. Ferreira, Icaro A. Marques, Wesley A. Conceição, Bruno Macedo, Clarice S. Machado, Alessandra Mascarello, Louise Domeneghini Chiaradia-Delatorre, Rosendo Augusto Yunes, Ricardo José Nunes, Andrew G. Hughson, Lynne D. Raymond, Pedro G. Pascutti, Byron Caughey, Yraima Cordeiro

**Affiliations:** 1 Faculdade de Farmácia, Universidade Federal do Rio de Janeiro, Rio de Janeiro, Rio de Janeiro, Brazil; 2 Instituto de Biofísica Carlos Chagas Filho, Universidade Federal do Rio de Janeiro, Rio de Janeiro, Rio de Janeiro, Brazil; 3 Departamento de Química, Universidade Federal de Santa Catarina, Florianópolis, Santa Catarina, Brazil; 4 Laboratory of Persistent Viral Diseases, Rocky Mountain Laboratories, National Institute of Allergy and Infectious Diseases, National Institutes of Health, Hamilton, Montana, United States of America; University of Melbourne, Australia

## Abstract

The prion protein (PrP) is implicated in the Transmissible Spongiform Encephalopathies (TSEs), which comprise a group of fatal neurodegenerative diseases affecting humans and other mammals. Conversion of cellular PrP (PrP^C^) into the scrapie form (PrP^Sc^) is the hallmark of TSEs. Once formed, PrP^Sc^ aggregates and catalyzes PrP^C^ misfolding into new PrP^Sc^ molecules. Although many compounds have been shown to inhibit the conversion process, so far there is no effective therapy for TSEs. Besides, most of the previously evaluated compounds failed *in vivo* due to poor pharmacokinetic profiles. In this work we propose a combined *in vitro*/*in silico* approach to screen for active anti-prion compounds presenting acceptable drugability and pharmacokinetic parameters. A diverse panel of aromatic compounds was screened in neuroblastoma cells persistently infected with PrP^Sc^ (ScN2a) for their ability to inhibit PK-resistant PrP (PrP^Res^) accumulation. From ∼200 compounds, 47 were effective in decreasing the accumulation of PrP^Res^ in ScN2a cells. Pharmacokinetic and physicochemical properties were predicted *in silico*, allowing us to obtain estimates of relative blood brain barrier permeation and mutagenicity. MTT reduction assays showed that most of the active compounds were non cytotoxic. Compounds that cleared PrP^Res^ from ScN2a cells, were non-toxic in the MTT assay, and presented a good pharmacokinetic profile were investigated for their ability to inhibit aggregation of an amyloidogenic PrP peptide fragment (PrP^109–149^). Molecular docking results provided structural models and binding affinities for the interaction between PrP and the most promising compounds. In summary, using this combined *in vitro*/*in silico* approach we have identified new small organic anti-scrapie compounds that decrease the accumulation of PrP^Res^ in ScN2a cells, inhibit the aggregation of a PrP peptide, and possess pharmacokinetic characteristics that support their drugability. These compounds are attractive candidates for prion disease therapy.

## Introduction

Transmissible spongiform encephalopathies (TSEs) are fatal neurodegenerative diseases that affect humans and other mammals [1], [2]. These diseases are characterized by cognitive and motor dysfunctions, and patients usually die within thirteen months after the onset of clinical symptoms [3]. TSE development is triggered by the conversion of native prion protein (PrP) into a misfolded form, named scrapie PrP (PrP^Sc^) [1]. While PrP^C^ is α-helix-rich and normally anchored through a GPI tether to the cellular surface, PrP^Sc^ has higher β-sheet content and deposits as insoluble aggregates in the intracellular and extracellular spaces [1], [2]. Comprehension of the molecular mechanisms responsible for PrP^C^ conversion into PrP^Sc^ is still not fulfilled. The protein-only hypothesis postulates that PrP^Sc^ is solely responsible for inducing misfolding and further conversion of newly synthesized PrP molecules into the abnormal conformation [4]; thus, PrP^Sc^ formation is amplified, characterizing this protein as a protein-only pathogen, capable of replication without the need of a coding nucleic acid molecule [4], [5]. However, the key participation of other cellular factors besides PrP^Sc^, such as glycosaminoglycans, nucleic acids, and lipids has been implicated in the conversion process [6], [7]. Understanding the conversion, the factors that are important for this event, and how to block or delay this process, may help developing therapeutic strategies for prion diseases.

To date, there is no effective therapy for TSEs. A great number and variety of molecules have been evaluated both *in vitro*, and *in vivo* for anti-scrapie activity [8]–[14]. Many small organic compounds block PrP conversion in cell cultures infected with scrapie prion strains [8], [9], and examples of several classes of inhibitors are known to prolong the lives of infected rodents *in vivo* [8]–[11], [13]. However, clinical applicability of these compounds is severely limited by a lack of activity when administered after the onset of clinical signs of disease, poor bioavailability to the brain, and/or high toxicity [11]–[13], [15]. Molecules assayed for prion disease treatment range from large organic molecules, such as polyanionic compounds, immunotherapeutics, and β-breaker peptides, to inorganic molecules, such as copper ions [9]. Among small organic compounds, there is a distribution along several different chemical classes, but a common feature is that practically all of the effective *in vitro* compounds were homo- or heterocyclic organic molecules with more than 2 rings [9], [11], [16]. Specially, quinoline and acridine derivatives have been shown to be effective in cell culture assays and to inhibit aggregation of prion protein domain *in vitro* [17]–[21]. These results suggest that the common structure of these organic molecules might be valuable as prototype compounds for TSE treatment. Unfortunately, a clinical trial (PRION-1) performed with quinacrine, an antimalarial compound found to present high anti-prion activity in cell models [17], [18], failed to present statistical results between patients receiving or not the drug [22]. Clinical trials for prion diseases are difficult to implement, as the number of individuals affected with TSEs is low each year (∼1:1,000,000 cases per year), and there are different forms of the disease [23]. Based on the lack of *in vivo* activity and the high toxicity of the compounds that entered clinical trials, the search for new compounds with reduced toxicity and increased efficacy is still greatly needed. Moreover, the mechanism(s) of action of anti-scrapie molecules is not clear. Some compounds may interact directly with PrP^C^, preventing its conversion into PrP^Sc^; while others may increase degradation of PrP^Sc^ by inducing its unfolding. Alternatively, inhibition may not involve direct interactions with either PrP^C^ or PrP^Sc^ [24], but instead be due to effects such as the stimulation of autophagy [25], a change in the pH of endocytic vesicles [17], or relocation of PrP^Sc^ into lysosomes [26], which, in turn, could increase the clearance of misfolded PrP.

**Figure 1:**
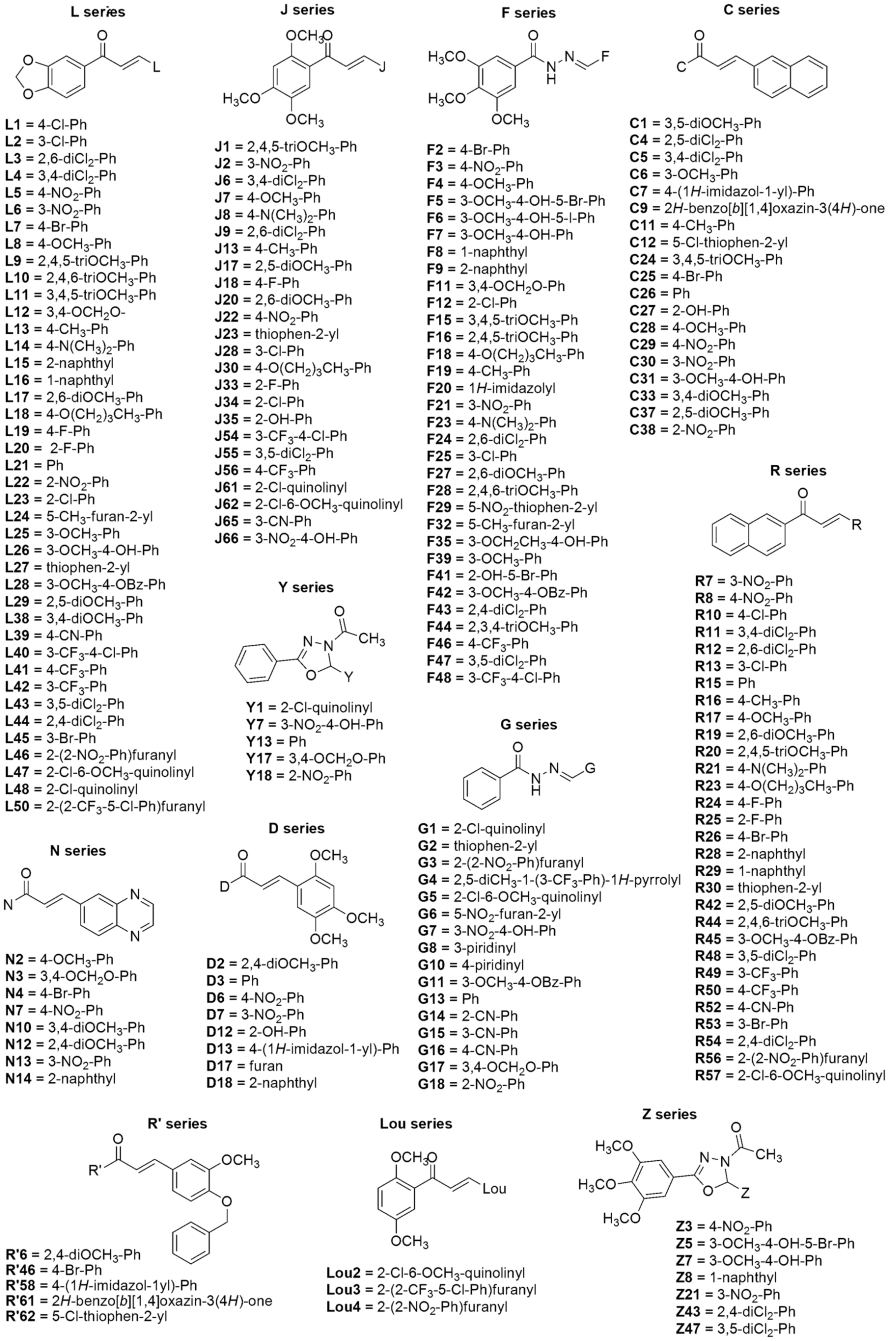
Chemical structure of the compounds investigated. Each series is formed by a group of compounds, as follows: C (19), D (8), F (31), G (11), J (36), L (41), Lou (3), N (8), R (30), R' (5), Y and Z (6). Ph  =  phenyl; Bz  =  benzyl.

Herein, we used a combined *in vitro*/*in silico* approach to evaluate the anti-scrapie activity of a library of ∼200 aromatic organic compounds belonging to different chemical classes, such as acylhydrazones, oxadiazoles, and chalcones [27]–[32] (Fig. 1). We identified compounds that were active in reducing accumulation of PrP^Res^ in a high-throughput assay with scrapie-infected N2a cells (ScN2a) cells. Some of those were also non-toxic to cells in culture as well as by *in silico* prediction. Direct interaction of active compounds with PrP was suggested by *in vitro* aggregation assays with PrP^109–149^ and by molecular docking. Based on the presented results, we propose that a group of chalcones and oxadiazoles are effective at PrP^Res^ reduction, and present acceptable pharmacokinetic profiles. Some of the compounds might be further evaluated in rodent models for prion disease, providing new alternatives for future TSE therapy.

## Materials and Methods

### Compounds/Chemistry

The acylhydrazones (F and G series), oxadiazoles (Y and Z series), and chalcones (C, D, J, L, Lou, N, R' and R series) (Fig. 1) were synthesized as previously reported [27]–[32] and characterized by melting points, infrared and nuclear magnetic resonance of ^1^H and ^13^C (data not shown). The compounds were solubilized in 100% dimethyl sulfoxide (DMSO) to 10 mM final concentration. Stock solutions in DMSO were further diluted in sterile H_2_O or PBBS (phosphate buffered saline, glucose and phenol red), pH 7.3, before performing the spectroscopic or cellular assays, respectively.

### PrP^109–149^ peptide

The PrP^109–149^ peptide was synthesized in solid phase and purified by RP-HPLC by GeneMed Synthesis, Inc. (San Antonio, TX, USA) with 90.35% final purity. Peptide identification was done by mass spectrometry analysis (MALDI-TOF). This peptide corresponds to a loop and the first α-helix of the N-terminal region of PrP [33]. This domain promptly aggregates when diluted in aqueous solutions at low pH (<6.0), as previously described [21]. PrP^109–149^ aggregation was monitored as function of time after dilution of a stock solution of the peptide (in 6 M urea, 10 mM SDS, 50 mM MES buffer, pH 5.0) in 50 mM [2-(*n*-morpholino)ethanesulphonic] (MES) buffer at pH 5.0 by monitoring light scattering values at 450 nm, upon illumination at the same wavelength.

### Cell lines

Prion-infected neuronal cell lines ScN2a [34] were grown as previously described [35]. Briefly, ScN2a cells infected with RML strain were split upon reaching confluence, and applied to a 96-well plate. After adhering to the bottom of the wells, the compounds were applied at varying concentrations (from 0.1 to 10 μM) to each well (in triplicate or quadruplicate) and cells were grown for 4 days in Opti-MEM (Gibco Life Technologies) supplemented with 2 mM glutamine, 10% FBS, penicillin and streptomycin. Murine neuroblastoma cell line Neuro-2a (N2a) was purchased from the Cell Bank of Rio de Janeiro, Brazil (Banco de Células do Rio de Janeiro/UFRJ) (code # CR098) and grown as previously described [36].

### Dot-blot assay

The protocol was done as described [35]. After incubation with the compounds for 4 days, ScN2a cells were analyzed by optical microscopy to discard wells with evident cell damage/loss. Medium was removed by suction and 50 μL of lysis buffer (5 mM Tris, 150 mM NaCl, 0.5% Triton X-100, and 0.5% sodium deoxycholate) was added to each well. After benzonase (Sigma-Aldrich) addition at 13.5 U/mL (stock solution: 324 U/μL), the plate was incubated at 37°C for 30 min. Proteinase K (PK) (Calbiochem) was added to each well at 0.025 mg/mL and incubated for 1 h at 37°C, and 200 μL of Pefabloc at 1 mM (Boehringer Manheim) was added to each well to inhibit the PK. A PVDF membrane (was prepared Immobilon-P, Millipore) by soaking in methanol, washing with H_2_O and further washing and equilibrating in TBS (tris-buffered saline, 50 mM Tris-Cl, pH 7.5, 150 mM NaCl). The membrane was mounted in the dot-blot apparatus (Minifold-1, Schleicher & Schuell BioScience GmbH), the wells were washed with TBS and the content of the 96-well plate was applied to the membrane with the help of a vacuum system. The membrane was removed from the dot-blot apparatus, incubated in 3 M guanidine isothiocyanate (GdnSCN) for 8 min at 25°C in TBS for exposure of PrP epitopes, washed 4 times with TBS, incubated for at least 1 h with the primary antibody (R30) diluted 1:5,000 in TBS-T (TBS with 0.05% Tween) plus 5% non-fat milk. After washing with TBS-T, the secondary antibody was added at 1:5,000 dilution, incubated for at least 1 h, the membrane was washed with TBS-T, AttoPhos® reagent (Promega) was added, membrane was left to dry prior to being read in a Typhoon scanner (GE healthcare). Intensity density for each well was quantified with the program ImageJ (http://imageJ.nih.gov/ij).

### RT-QuIC assay

Real time quaking-induced conversion was done as follows based on previously published protocol [37]. Briefly, normal brain homogenate (NBH) or brain homogenate from hamsters clinically ill with the 263 K scrapie strain were used to seed the conversion reaction, using hamster recombinant PrP^90–231^ as the PrP^Sen^ (PK-sensitive PrP) conversion substrate in the presence of 300 mM NaCl. Selected compounds were applied at 25 or 50 μM final concentration and the clear bottom 96-well plate was incubated for ∼20 h at 42°C in a double orbital fluorescence reader FluoStar OPTIMA (BMG LabTech). Thioflavin T (Th-T) fluorescence emission (excitation: 450±10 nm, emission: 480±10 nm) was followed over time as the experimental read-out.

### Spectroscopy

Light scattering (LS) and fluorescence measurements were performed in a Jasco FP 6300 spectrofluorimeter (Jasco Corp., Tokyo, Japan). For the aggregation kinetics assay, PrP^109–149^ previously stored in a solution with 6 M urea and 10 mM SDS at pH 5.0 was diluted to 5 μM final concentration in 50 mM MES buffer at pH 5.0 (positive control). To verify whether the compounds were able to inhibit the peptide aggregation, PrP^109–149^ was incubated in the presence of varying concentrations of the compounds and LS was collected from 430 to 470 nm upon illuminating the samples at 450 nm. The LS of PrP^109–149^ in 6 M urea was used as negative control, since in this condition the peptide does not aggregate.

### MTT reduction assay

N2a cells (up to 80% confluence) were plated into 96-well plates in complete cell media (DMEM) at a density of ∼5,000 cells/well and were incubated overnight at 37°C in a 5% CO_2_ atmosphere. Compounds stock solutions were diluted to 500 μM in sterilized water and then further applied to the cells at final concentrations of 1, 5, or 10 μM. After 72 h incubation, MTT (3-[4,5-dimethylthiazol-2-yl]-2,5-diphenyl tetrazolium bromide) reduction was evaluated as previously described [36].

### Molecular Docking

Prior to the docking procedure, the three-dimensional structures of the compounds were obtained with AVOGADRO software [38], in which energy minimization is calculated by a conjugated gradient algorithm [39]. Docking between globular domains (residues ∼125 to 230) of recombinant prion protein (rPrP) from mouse (PDB: 1AG2) and compounds was carried with SWISSDOCK web server based on EADock DSS [40], and calculations were made with CHARMM force field [41] on external computers from the Swiss Institute of Bioinformatics. Molecular complexes are ranked by the most favorable binding energies, and we selected among those one structure representing the best binding mode, based on an energy average value corresponding to the first five ranked structures. This procedure allows obtaining successful docking for ligands possessing <10 rotating bonds [42], such as those used in this work.

### *In silico* physicochemical and pharmacokinetics analysis

Physicochemical and pharmacokinetic (PK) parameters, as well as overall drug-scores for selected compounds were predicted *in silico* using the software ChemSilico (http://http://www.chemsilico.com) [43], Advanced Chemistry Development, Inc. (ACD/Labs, ACD/Percepta Platform, version 12.01, Toronto, ON, Canada, www.acdlabs.com, 2013), and Osiris Property Explorer (http://www.organic-chemistry.org/prog/peo). Three-dimensional structures of the compounds were constructed as described above, and ChemSilico parameters as mutagenicity (CSMIA) and ability of the drug to be absorbed passively by membranes (CSHIA) were determined. Osiris predicted if compounds were mutagenic or not. ACD/Labs Percepta Platform parameters as LogP (an estimate of the value of the octanol-water partitioning coefficient that provides the propensity of a molecule to insert into lipophilic membranes), brain/plasma equilibration rate (log(PS*f_u,brain_)) and brain penetration were also determined. Log(PS*f_u,brain_) is a combination of permeation rate and fraction unbound of the compound in brain. Brain penetration classifies capability of the compound to access the central nervous system. ACD/Percepta classifies compounds as sufficiently permeable in the CNS to be active in CNS (S) or inactive (I) due to low penetration. Besides, prediction of mutagenicity was also done with ACD/Percepta, yielding the probability of a positive result in the Ames test. Bioavailability parameter that predicts the fraction of the specified drug dose that reaches circulation after oral administration was also obtained. The utilized software compare the compounds with known molecules and predict some properties based on their quantitative-structure-activity-relationship (QSAR).

### Statistics

Graph Pad Prism was used for statistical analyses of the data. One-Way ANOVA Tukey test was used to determine significant differences between controls and treatment with the compounds (*P<0.05, **P<0.01, ***P<0.001). NS means non-significant. Data shown are the mean of triplicates or quadruplicates, and error bars show standard error (SEM), or are representative of at least two independent experiments.

### Ethics statement

Rocky Mountain Laboratories is an Association for Assessment and Accreditation of Laboratory International Care (AAALAC) accredited facility, and all animal procedures were carried out in strict accordance with the recommendations in the Guide for the Care and Use of Laboratory Animals of the National Institutes of Health. The protocol was approved by the institution's Animal Use and Care Committee and the National Institutes of Health (Protocol Number: 2010–45).

## Results and Discussion

**Figure 2:**
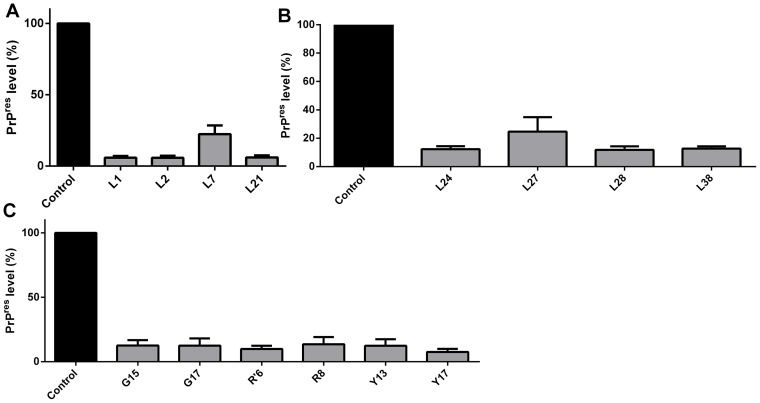
Decrease of PrP^Res^ levels in ScN2a cells. ScN2a cells were treated with the compounds belonging to the L (panels A and B), G, R', R and Y (panel C) series at 10 µM. After four days of incubation with the compounds, cells were lysed, treated with PK and PrP^Res^ was detected in the dot-blot assay with anti-PrP antibody (R30). Control bar (medium) represents intensity density of the blot from wells without compound addition, corresponding to 100% of PrP^Res^ content. Quantification of the assay was done by integration of the density of each dot using ImageJ software. All bars had * *P*<0.05 in relation to control.

**Figure 3:**
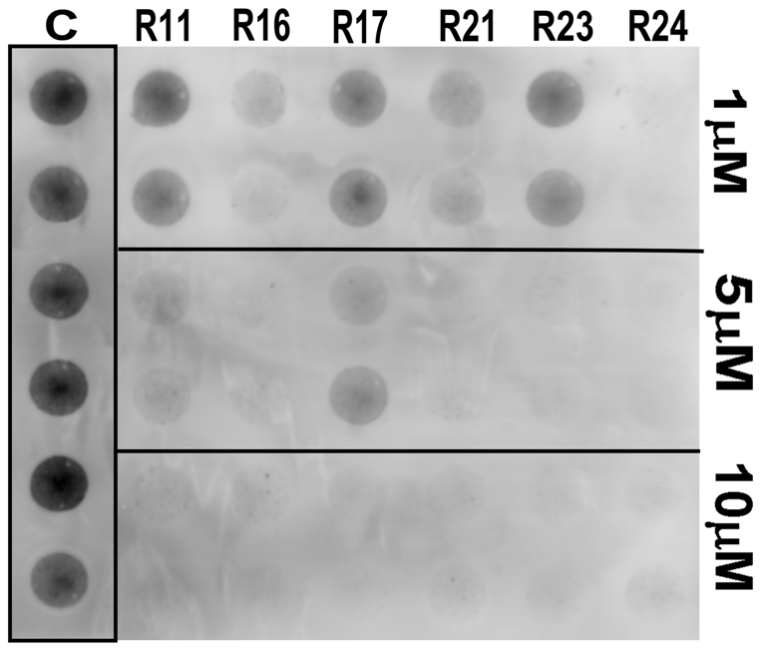
Representative dot-blot showing PK-resistant PrP (PrP^Res^) accumulated in ScN2a cells grown in the presence of compounds. ScN2a cells were grown for 4-well plates in the presence of compounds from the R series at 1, 5, and 10 μM final concentrations. Cell lysates were subjected to PK treatment and dot-blotting with antiserum R30. Control wells (C) show untreated cells.

**Figure 4:**
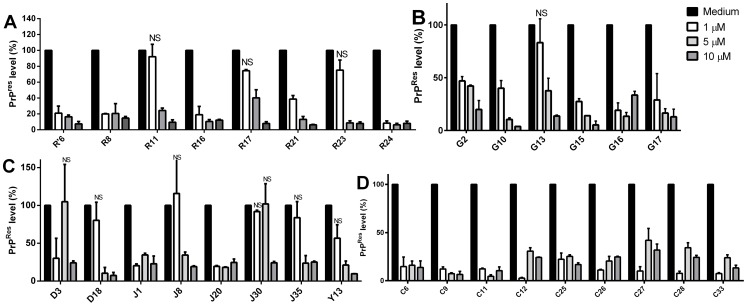
Dose-response curves showing PrP^Res^ accumulated in ScN2a cells grown in the presence of selected compounds. ScN2a cells were treated with compounds from the R and R' (panel A); G (panel B); D, J and Y (panel C); and C series (panel D) at 1, 5, or 10 µM. Control bar (medium) represents intensity density of the blot from wells without compound addition, corresponding to 100% of PrP^Res^ content. Quantification of the assay was done by integration of the density of each dot using ImageJ software considering as control the final DMSO concentration in each well. All bars had * *P*<0.05 in relation to control except those labeled as NS (non-significant).

To select the most promising compounds we screened their anti-scrapie activity in neuroblastoma cells in culture persistently infected with the RML prion strain (ScN2a) in a high-throughput assay. This assay allows assessment of a great number of compounds by following their effects on the accumulation of proteinase-K resistant PrP (PrP^Res^) in the ScN2a cell cultures grown in 96-well plates [35], [44]. Cells were grown in the presence of the compounds during four (4) days. After this incubation time, each well was inspected by optical microscopy to detect any evident cell damage or death. Compounds that clearly reduced cell number were not further investigated (Table S1). To estimate the relative PrP^Res^ accumulation in treated and untreated cultures, we treated cell lysates with proteinase K to eliminate PrP^C^, and subjected them to dot-blot analysis using an anti-PrP antibody. We initially screened the compounds at a fixed concentration (10 μM) (only representative compounds are shown in Fig. 2), and those active in reducing PrP^Res^ signal were then tested in ScN2a cells at concentrations ranging from 1 to 10 μM; a representative blot is shown in Fig. 3. Dose-response curves were obtained by integrating the density of each blot (Fig. 4).

**Figure 5:**
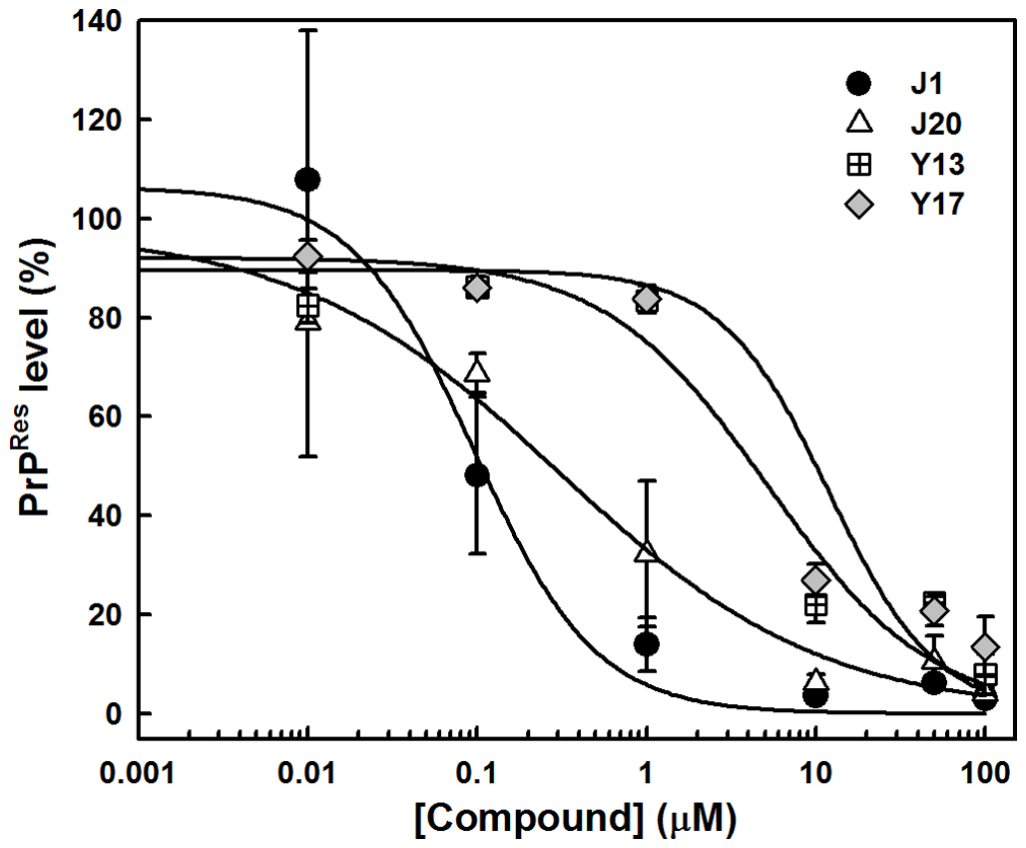
Dose-response curves for compounds from the J and Y series. Lysates of ScN2a cells grown in the presence of the compounds for 4-blotted as described in the Methods section. Quantification of the relative dot-blot signal intensities were done with ImageJ considering as the control the final DMSO concentration in each well. The curves were fitted by a sigmoidal curve with SigmaPlot software v. 10.0.

From the ∼200 homo- and heterocyclic organic compounds evaluated, belonging to different chemical classes, we obtained 47 compounds effective in reducing PrP^Res^ labeling in the dot-blot assay (Figs. 2, 3 and 4; Table S1). Among the latter, ∼20 were effective at 1 μM in reducing PrP^Res^ levels >50%. Dose-response curves with selected compounds from the J and Y series that were effective in the dot-blot assay were done with a broader concentration range (Fig. 5). The fitting of the curves yielded approximate IC_50_ values <1 μM for J1 and J20, and <10 μM for Y13 and Y17 (Fig. 5).

With compounds that were apparently non-toxic and effective in the blot assay (at concentrations ranging from 1 to 10 μM), we performed *in silico* predictions of pharmacokinetics and drugability of the compounds using ACD/Percepta Platform, Osiris, and ChemSilico [43] software (Table 1). An ability to cross the blood brain barrier (BBB) is a key asset for compounds to be used for therapies of TSEs or other central nervous system afflictions. In addition, little or no hepatotoxicity is desired for orally delivered drugs. ACD/Percepta Platform calculated the water/octanol partition coefficient (LogP) of the compounds, providing estimates of the lipophilic or hydrophilic characteristics of the compounds. The brain/plasma equilibration rate (log(PS*f_u,brain_)) and brain penetration values were also estimated by ACD/Percepta. Compounds were classified as sufficiently permeable in the CNS (S) or inactive (I) due to low penetration (Table 1; Table S1). Bioavailability for each compound was also predicted. Mutagenic propensity was obtained with ACD/Percepta and Osiris, as well as by ChemSilico that ranked the substances as mutagenic or non-mutagenic, based on their ability to induce DNA damage (CSMIA). Osiris and ACD/Percepta provide these results by comparing the structures of investigated compounds with those of other molecules in the database that are known to be mutagenic. ChemSilico also predicted the percent of human intestinal absorption (CSHIA).

**Table 1:** *In silico* prediction of physicochemical and pharmacokinetic properties.

*Compound*	*LogP* a	Log (PS*f_u,brain_) a	*BP* a	*Ames Test (%)* a	*Bioavailability (%)* a	*CSMIA^b^*	*CSHIA (%)^b^*	*Mut.^c^*
C4	5.52	−3.3	I	19	30–70	1	79.0	+/−
C6	5.52	−3.1	S	15	30–70	1	84.3	Yes
C9	3.7	−2.7	S	24	30–70	1	90.8	Yes
C11	5.38	−3.2	S	15	30–70	1	82.9	Yes
C25	5.39	−3.2	S	16	30–70	1	78.9	Yes
C26	4.56	−3	S	15	30–70	1	73.7	Yes
C27	4.67	−3.1	S	29	30–70	1	73.7	+/−
C28	4.7	−3.1	S	15	30–70	1	86.6	Yes
C29	4.51	−3	S	84	30–70	1	84.4	Yes
C33	4.14	−3	S	25	30–70	1	86.6	Yes
D3	3.26	−2.4	S	29	>70	n.d.	n.d.	Yes
D18	4.21	−2.9	S	40	30–70	1	85.3	Yes
G2	3.34	−2.5	S	36	>70	1	92.7	No
G10	2.57	−2.3	S	41	>70	1	91.2	No
G13	2.99	−2.4	S	30	>70	1	94.0	No
G15	2.99	−2.4	S	34	>70	1	93.0	+/−
G16	2.95	−2.4	S	34	>70	1	93.0	Yes
G17	2.95	−2.4	S	28	>70	1	93.2	No
J1	2.99	−2.5	S	59	>70	0	78.4	No
J8	2.99	−2.6	S	46	30–70	1	92.4	Yes
J20	3.48	−2.6	S	27	30–70	0	83.1	No
J35	3.48	−2.4	S	39	>70	0	87.0	No
L1	3.43	−2.5	S	20	30–70	1	85.7	Yes
L2	3.43	−2.5	S	20	30–70	1	85.8	Yes
L7	3.43	−2.5	S	20	30–70	1	84.8	Yes
L21	3.04	−2.3	S	18	30–70	1	n.d.	Yes
L24	2.57	−2.2	S	22	>70	1	n.d.	Yes
L27	2.67	−2.2	S	16	>70	1	n.d.	Yes
L28	4.27	−2.9	S	24	30–70	1	n.d.	Yes
L38	2.60	−2.3	S	18	>70	1	n.d.	Yes
Ŕ6	4.91	−3.2	S	37	30–70	1	n.d.	No
R8	4.51	−3.1	S	84	30–70	1	n.d.	Yes
R11	5.83	−3.3	I	15	30–70	1	n.d.	Yes
R13	5.23	−3.2	S	16	30–70	1	n.d.	Yes
R16	5.38	−3.2	S	15	30–70	1	n.d.	Yes
R17	4.7	−3.1	S	15	30–70	1	84.3	Yes
R21	4.92	−3.1	S	28	30–70	1	93.0	Yes
R23	5.72	−3.1	S	13	30–70	1	84.0	Yes
R24	4.76	−3.1	S	17	30–70	1	81.4	Yes
R26	5.39	−3.3	S	16	30–70	1	79.0	Yes
R28	5.85	−3.3	I	19	30–70	1	77.5	Yes
R29	5.97	−3.3	I	9	30–70	1	77.7	Yes
R49	5.97	−3.3	I	11	30–70	1	87.4	Yes
Y13	2.74	−2.2	S	26	>70	n.d.	n.d.	No
Y17	2.74	−2.2	S	24	>70	1	94.3	No

^a^ ACD/Labs Percepta Platform, ^b^ChemSilico, ^c^Osiris predictions. Log (PS*f_u,brain_) is a combination of permeation rate and fraction unbound in brain. BP (Brain Penetration) classifies compounds as sufficiently CNS-permeable to be active (S) or inactive (I) in the CNS. Ames test shows the probability of a mutagenic positive result in this assay. Bioavailability predicts the fraction of the specified drug dose that reaches circulation after oral administration. CSMIA is a predictor of mutagenicity (ability to induce DNA damage) based on the Ames test (1, mutagenic; 0, non-mutagenic). CSHIA (human intestinal absorption predictor) estimates the % of passive absorption of the compound through membranes. Mutagenicity (Mut.) estimates if a compound is mutagenic or not, by comparing the structure of the compound with molecules deposited in the Osiris database.

From 45 compounds tested, only J1, J20, and J35 were predicted to be non-mutagenic by both ChemSilico and Osiris (Table 1). ACD/Percepta platform provided a 59% probability of mutagenicity for J1. Compounds from all other series were predicted to be mutagenic by ChemSilico. On the other hand, Osiris and ACD/Labs predicted, in addition to J1, J20 and J35, that compounds G2, G10, G13, G17, Y13 and Y17 would be low or non-mutagenic (Table 1). Compounds from the G and Y series, J1 and J35, besides being predicted to be permeable to the CNS, were also estimated to have high bioavailability (>70%) by ACD/Labs (Table 1). Together with the above mentioned compounds, some compounds from the D, L, and R series were predicted to be permeable to the CNS, in addition to have a low probability to be mutagenic (20–30%), as estimated by ACD/Labs (Table 1). In agreement with these results these compounds presented positive LogP values showing that they are lipophilic, and for this reason, tend to distribute into hydrophobic compartments such as lipid bilayers of cellular membranes. CSHIA parameter predicts the capacity of compounds to be absorbed passively through membranes by paracellular or transcellular pathways. This parameter predicted that all possibly non-mutagenic compounds would be highly orally absorbed (>80%), in agreement with bioavailability data provided by ACD/Percepta (Table 1). Estimated human jejunum permeability (cm/s) and the intestinal passive absorption rate (Ka) values resided between 7.3–8.9×10^−4^cm/s and 0.052–0.061 min^−1^, respectively (data not shown) for all compounds. Most compounds from the C and R series (chalcones) were predicted as mutagenic by Osiris and ChemSilico; thus, these compounds were not further investigated. Taken together, these results suggest that J1, J20 and J35 (and possibly D3, D18, G2, G10, G13, G17, Y13 and Y17) are potential drug candidates. Thus, we pursued further *in vitro* investigations of the activities and mechanisms of action of these compounds.

**Figure 6:**
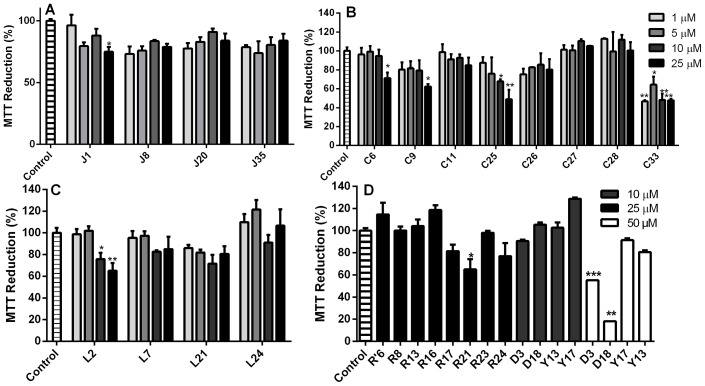
Cellular dysfunction evaluated by MTT reduction in the presence of selected compounds. Dose-response results for compounds from the J (panel A), C (panel B), L (panel C), and R, R', Y and D series (panel D) applied to neuroblastoma (N2a) cells in 96-well plates. Compounds were added to N2a monolayers at final concentrations of 1, 5, 10, 25 and/or 50 μM. After 72 h, MTT reduction was evaluated as described in the Material and Methods section. Data are expressed as the percentage of MTT reduction in relation to the control (0.25% DMSO in complete cell media). Error bars represent standard deviations of at least three independent measurements, each one in triplicate. *P<0.05; **P<0.01; ***P<0.001.

To verify whether the decrease in the PrP^Res^ content observed in the dot-blot assay resulted from reduced cellular viability, we followed MTT reduction by non-infected N2a cells [36], [45] in the presence of selected compounds from the J, G (not shown), D, C, L, R and R' (investigated due to high PrP^Res^ reduction activity in the dot-blot assay), and Y series (Fig. 6; Table S1). The majority of the assayed compounds did not cause significant cell dysfunction when applied to N2a cells at up to 25 μM, which exceeded the effective concentrations of ≤10 μM) (Fig. 6). These data show that most of the compounds are not substantially toxic to cultured cells, except L2 (panel C), C25 and C33 (panel B), and G2, G10, G15 and G17 (not shown) that were toxic at lower concentrations (Fig. 6). Based on the MTT reduction data and the predicted mutagenic profile, compounds from the C series were not further investigated.

Compounds that were effective in reducing PrP^Res^ levels in ScN2a cells without being cytotoxic may act by either increasing degradation of PrP^Res^ or by depleting PrP^C^ from the cells (either by increasing its turnover, or by reducing its expression), thereby reducing the pool of substrate for conversion into PrP^Res^.

**Figure 7:**
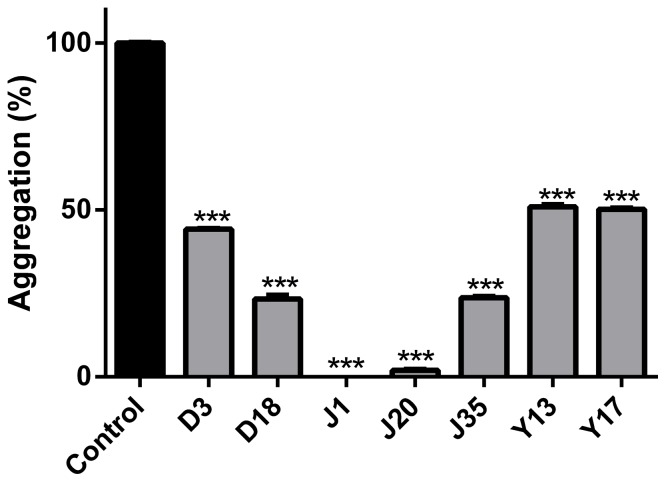
Anti-aggregating effects of compounds from J, D and Y series followed by PrP^109–149^ aggregation assay. PrP^109–149^ at 5.0 μM was incubated in the presence of selected compounds (effective in reducing PrP^Res^ levels in ScN2a cells) at 25 μM and light scattering (LS) was monitored at 450 nm. Control bar (100% aggregation) corresponds to peptide diluted in 50 mM MES buffer only (pH 5.0) with subtraction of LS values for PrP^109-149^ diluted in 6 M urea, a condition in which the peptide is completely unfolded and non-aggregated. ***P<0.001.

Our next approach was to determine whether compounds from the D (3, 18), J (1, 20, 35), and Y (13, 17) series were able to inhibit *in vitro* aggregation of an amyloidogenic synthetic PrP peptide. Since the compounds Y13 and Y17 were considered non-mutagenic by Osiris and ACD/Labs, and were not significantly cytotoxic up to 50 μM (Fig. 6D), they were included in this assay. Although compounds D3 and D18 were cytotoxic at 50 μM (but not at 10 μM) (Fig. 6D), they were also predicted to be drugable by ACD/Labs, and were effective in the dot-blot assay (Fig. 4); thus, these compounds were evaluated in the aggregation assay. The prion domain utilized was the PrP^109–149^ region that has been previously used by our group as a model for following rapid protein aggregation and to screen for anti-aggregating compounds [21], [46], [47]. This peptide spans from within the flexible N-terminal domain into the beginning of α-helix 1 of recombinant PrP^C^ [33]. It is predicted to participate in the α-helix to β-sheet conversion when PrP^Sc^ is formed [48]. Although its aggregation does not possess a lag phase that is typical of spontaneous amyloid fibril formation [49], its rapid oligomerization allows verification of the effect of compounds that modulate this process, indicating that such compounds interact with this PrP domain. The peptide is kept in an unfolded conformation (6 M urea, pH 5.0), and upon dilution into buffer free of urea (MES, pH 5.0), light scattering values increase, indicating formation of larger particles (aggregation). We found that the assayed compounds, which blocked PrP^Res^ accumulation in RML-infected ScN2a cells (Figs. 4 and 5), and were predicted to be non-mutagenic by ChemSilico, Osiris and/or ACD/Labs software (Table 1), also reduced PrP^109–149^ aggregation in a concentration range similar to that which blocked PrP^Res^ accumulation (Fig. 7). To note, J1 and J20 completely blocked PrP^109–149^ aggregation. Therefore, we have evidence that these PrP^Res^ inhibitors can bind at least to recombinant PrP peptide.

**Figure 8:**
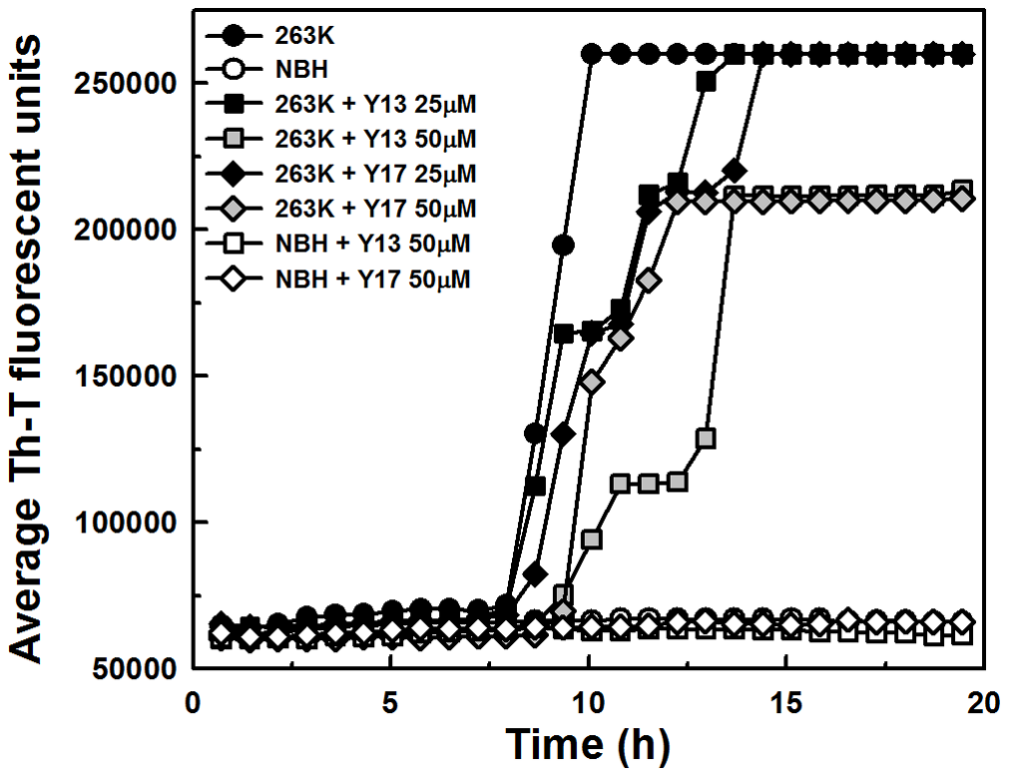
Evaluation of the compounds' capacity to delay PrP^Sen^ conversion into PrP^Res^ by RT-QuIC assay seeded with 263 K scrapie. RT-QuIC reactions were seeded with 10fg of 263 K infected hamster brain or the equivalent amount of normal brain homogenates (NBH). The substrate for the reaction was recombinant hamster PrP^90–231^. NaCl was used at 300 mM final concentration. Compounds Y13 and Y17 were assayed at 25 and 50 μM. The assay was followed by ThT fluorescence (excitation 450 nm; emission 480 nm) emission over time (average of 4 replicate wells).

The real time quaking induced conversion (RT-QuIC) assay [37], [50] was performed to evaluate the capability of some of compounds to directly inhibit cell-free conversion of recombinant PrP^C^ (sensitive to PK digestion) into PrP^Res^. The basis of the assay is to seed a conversion reaction with infected scrapie brain homogenate containing low amounts (fg) of PrP^Res^ using recombinant PrP^C^ as a substrate [37], [50]. Th-T fluorescence emission is followed over time, while the 96-well reaction plate is incubated at 42°C in a double orbital shaker. The 263 K prion strain was used to seed the conversion of recombinant hamster PrP^90–231^ in the presence of the compounds Y13 and Y17 that were effective in reducing PrP^Res^ levels and predicted to be non-mutagenic by Osiris and ACD/Labs (Figs. 4 and 5; Table 1). The compounds belonging to the J and D series, as well as other active compounds in previous assays were not evaluated by this methodology because they absorb in the same wavelength range as Th-T (400–460 nm). We found that, although none of the compounds could totally block PrP^Res^ formation when the reaction was seeded with 263 K prions, all increased the lag phase for conversion when added at 50 μM, suggesting that at least transient interaction with PrP was taking place (Fig. 8).

**Figure 9:**
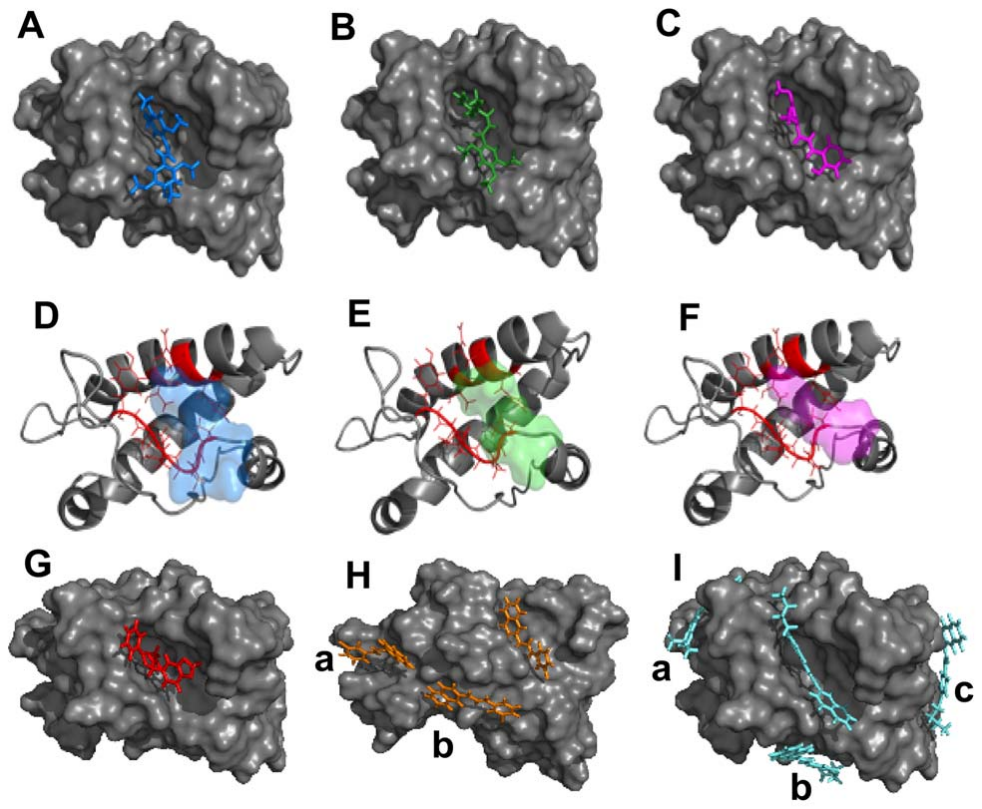
*In silico* prediction of interaction between murine PrP (1AG2.pdb) and anti-prion compounds realized by molecular docking. **A**, **B**, **C** and **G** represent the binding mode for compounds J1 (blue), J20 (green), J35 (magenta), and Y17 (red), respectively, in the same hydrophobic pocket of rPrP (gray). **D**, **E** and **F** are cartoon representations of rPrP secondary structure with residues directly participating in the interaction colored in red (P158, N159, Q160, V161, Y162, T183, Q186 and H187) and the compounds are shown as transparent surfaces in the same colors as in **A** (J1), **B** (J20) and C (J35). **H** and **I** show binding modes for compounds with binding stoichiometry >1:1 that are: R13 (orange) and R23 (cyan), respectively. **a**, **b** and **c** indicate binding modes outside the main binding pocket.

To further address if direct interaction with the prion protein would represent the mechanism of action of the compounds, we performed computational analysis with the globular domain of murine recombinant PrP (PDB entry: 1AG2) (Fig. 9, Table 2) and the compounds that were effective *in vitro*. Some compounds that had poor pharmacokinetic profile but were highly effective in the ScN2a assay and were non toxic in the MTT reduction assay (as some compounds from the R series) were also investigated. The 3D low energy conformation of the compounds was generated and molecular docking results indicated that all assayed compounds should interact with PrP with reasonable affinities (Table 2). Small PrP ligands with lower binding energies (<−11 kcal/mol) than those calculated here (<−6 kcal/mol) have been identified previously [51]. However, selected compounds investigated in the present work reduce PrP^Res^ levels to lower values and at lower concentrations than other reported PrP ligands [51]. These results led us to conclude that higher binding affinities for PrP are not essential for improved anti-scrapie activity.

**Table 2:** Energies and stoichiometry ratios for binding of recombinant murine PrP (1AG2) to compounds from D, G, J, L, R and Y series obtained from molecular docking.

*Compound*	*Energy, ΔG* *(kcal/mol)*	*Stoichiometry* *
D3	−6.67	1:1
D18	−7.09	1:1
G2	−6.19	2:1
G10	−6.42	1:1
G13	−6.46	1:1
G17	−6.56	1:1
J1	−7.31	1:1
J20	−7.02	2:1
J35	−6.50	1:1
L24	−6.54	1:1
L27	−6.61	1:1
L38	−6.99	1:1
R13	−6.41	3:1
R16	−6.45	1:1
R17	−6.64	1:1
R21	−6.60	1:1
R23	−6.82	4:1
R24	−6.44	1:1
R26	−6.71	2:1
Y13	−6.51	2:1
Y17	−6.76	1:1

Stoichiometry: compound:PrP molar ratio. Energy values are only shown for the main binding mode For compounds with binding stoichiometry >1:1.

Molecular docking provided mainly two binding modes (BMs), although stoichiometries higher than 2:1 (compound:PrP) were predicted (Table 2; Fig. 9). The most prevalent BM (pocket 1) was found between helix-2 and the antiparallel β-sheet of murine PrP, and no hydrogen bonds were identified; only hydrophobic contacts maintained the complex. Aminoacid residues identified in the pocket 1 were P158, N159, Q160, V161, Y162, T183, Q186 and H187 (Fig. 9). Interaction of PrP(121–231) with a quinoline heterocyclic compound was recently probed by NMR evidencing a similar binding region, with participation of residues 186 and 187 [52]. Curiously, taking into account that only residues 125 to 149 are present both in the PrP^109–149^ peptide and the 1AG2 structure, none of those were identified as possible binding sites for the investigated compounds. This result suggests that either there is another binding site (N-terminal) for the compounds along the PrP structure (e.g., as suggested for some inhibitor classes in reference 13), or that the peptide alone possesses a conformation that is not similar to its conformation in the full-length PrP structure, thus allowing interaction with the compounds only when the peptide is free in solution. Compounds that bind PrP^C^ directly, as those predicted here, may reduce PrP^C^'s availability to be converted into PrP^Res^, as found with pentosan polysulfate, and phosphorothioate oligonucleotides that cluster PrP^C^ and internalize it [53], [54]. A binding stoichiometry of 1:1 (compound:PrP) was calculated for most compounds; however, the compounds G2, J20, R26 and Y13 had 2:1 stoichiometries, and the compounds R13 and R23 were predicted to bind PrP at 3:1 and 4:1 molar ratios, respectively (Table 2; Fig. 9). Multiple binding modes were also shown for other small ligands for PrP by *in silico* prediction and surface plasmon resonance measurements [51]. Here, although predicted affinities (ΔG) were similar for all binding modes, interactions outside the main pocket (binding mode 1) occurred in superficial binding sites, i.e. in regions highly solvent-exposed, thus suggesting that these interactions are transient and, therefore, would not be crucial for anti-scrapie activity. The main binding mode predicted here shares similar location (residues from helix 2) with the binding region for specific PrP ligands [51], [52]. Experimental methodologies, such as isothermal titration calorimetry, and surface plasmon resonance (SPR), will allow validating these interactions and obtaining final thermodynamic and stoichiometric parameters.

Results with all investigated compounds (a total of 198) are summarized in the Table S1, evidencing the most promising compounds. In summary, we identified new compounds (chalcones and oxadiazoles) that were active in reducing accumulation of PrP^Res^ in ScN2a cells in the micromolar range, were non-toxic to cells as tested in culture, and had predicted pharmacokinetic profiles consistent with central nervous system bioavailability. The methoxychalcones J1, J20 and J35 and oxadiazoles Y13 and Y17 reduced PrP^Res^ levels in ScN2a cells by more than 50% in comparison with untreated cells at concentrations <1 μM (J1, J20), <5 μM (J35) and at ∼10 μM (Y13, Y17). Furthermore, these compounds significantly inhibited PrP^109–149^ peptide aggregation at micromolar concentrations and molecular docking results suggested their direct binding to PrP^C^. Our data indicate that these compounds can interact directly with PrP molecules but it remains to be determined what type of interactions are responsible for inhibiting PrP^Sc^ formation in scrapie-infected cells. Interactions with PrP^C^ might stabilize it and prevent its conversion into PrP^Sc^. Interactions with PrP^Sc^ might block binding of PrP^C^ at the seeding surface. In any case, we propose that compounds from the J (J1, J20 and J35) and Y (Y13 and Y17) series are attractive candidates for *in vivo* evaluation in rodent models for prion diseases.

## Supporting Information

Table S1 **Summary of all** ***in vitro*** **and** ***in silico*** **results evidencing the most promising compounds.**
(DOC)
