# Supplementary material for: Anti-Prion Activity of a Panel of Aromatic Chemical Compounds: In Vitro and In Silico Approaches
Source: PLoS One. 2014 Jan 6;9(1):e84531. doi: 10.1371/journal.pone.0084531 (PMC3882252; doi:10.1371/journal.pone.0084531)
Supplement: Table S1 — Summary of all in vitro and in silico results evidencing the most promising compounds. (DOC) [file pone.0084531.s001.doc]

**Table S1: Summary of all *in vitro* and *in silico* results evidencing the most promising compounds**

| **COMPOUND** | **Evident cell damage**  **(ScN2a cells)^a^** | **PrP-Res reduction in ScNa2 cells^b^** | **PK properties**  ***(in silico*)^c^** | **Physicochemical properties (*in silico*)^d^** | **Cytotoxicity**  **(N2a cells)^e^** | **Possibility to interact directly with PrP^f^** |
| --- | --- | --- | --- | --- | --- | --- |
| **C1, C31, C37, C38** | Yes | N.I. | N.I. | N.I. | N.I. | N.I. |
| **C5, C7, C24, C30** | No | No | N.I. | N.I. | N.I. | N.I. |
| **C25, C33** | No | Yes | I | S | Yes | Yes* |
| **C4, C6, C9, C11, C26, C27, C28, C29** | No | Yes | I | S | No | Yes* |
| **C12** | No | Yes | N.I. | N.I. | N.I. | N.I. |
| **D2, D6, D7, D12, D13, D17** | Yes | N.I. | N.I. | N.I. | N.I. | N.I. |
| **D3, D18** | No | Yes | I | S | No | Yes |
| **F2, F3, F4, F5, F6, F7, F8, F9, F11, F12, F15, F19, F21, F23, F24, F25, F27, F28, F29, F32, F39, F41, F42, F43, F44, F46, F47** | Yes | N.I. | N.I. | N.I. | N.I. | N.I. |
| **F16, F18, F20, F35, F48** | No | No | N.I. | N.I. | N.I. | N.I. |
| **G1, G3, G4, G7, G8, G18** | No | No | N.I. | N.I. | N.I. | N.I. |
| **G2, G10** | No | Yes | +/- | S | Yes | Yes* |
| **G5, G6** | Yes | N.I. | N.I. | N.I. | N.I. | N.I. |
| **G11, G14** | No | No | N.I. | N.I. | N.I. | N.I. |
| **G13** | No | Yes | +/- | S | No | Yes* |
| **G15, G17** | No | Yes | +/- | S | Yes | Yes* |
| **G16** | No | Yes | I | S | No | Yes* |
| **J1** | No | Yes | S | S | No | Yes* |
| **J2, J6, J9, J13, J17, J18, J22, J23, J28, J33, J34, J54, J55, J65** | Yes | N.I. | N.I. | N.I. | N.I. | N.I. |
| **J8** | No | Yes | I | S | No | N.I. |
| **J20** | No | Yes | S | S | No | Yes* |
| **J30** | No | Yes | N.I. | N.I. | N.I. | N.I. |
| **J35** | No | Yes | S | S | No | Yes* |
| **J7, J56, J61, J62, J66** | No | No | N.I. | N.I. | N.I. | N.I. |
| **L1** | No | Yes | I | S | N.I. | N.I. |
| **L2** | No | Yes | I | S | Yes | N.I. |
| **L3, L4, L5, L6, L9, L12, L13, L22, L23, L25, L40, L43, L44, L45, L46, L47, L50** | No | No | N.I. | N.I. | N.I. | N.I. |
| **L8, L10, L11, L14, L15, L16, L17, L18, L19, L20, L26, L29, L39, L41, L42, L48** | Yes | N.I. | N.I. | N.I. | N.I. | N.I. |
| **L7, L21, L24** | No | Yes | I | S | No | N.I. |
| **L27, L28, L38** | No | Yes | I | S | N.I. | N.I. |
| **Lou2, Lou3, Lou4** | Yes | N.I. | N.I. | N.I. | N.I. | N.I. |
| **N2, N3, N4, N10, N12, N13, N14** | Yes | N.I. | N.I. | N.I. | N.I. | N.I. |
| **N7** | No | No | N.I. | N.I. | N.I. | N.I. |
| **R7, R10, R12, R19, R25, R42, R44, R50, R52, R53, R54, R57, R’58** | Yes | N.I. | N.I. | N.I. | N.I. | N.I. |
| **R’6** | No | Yes | +/- | S | No | N.I. |
| **R8** | No | Yes | I | S | No | N.I. |
| **R11** | No | Yes | I | I | N.I. | N.I. |
| **R13** | No | Yes | I | S | No | Yes* |
| **R15, R20, R30, R45, R’46, R48, R56, R’61, R’62** | No | No | N.I. | N.I. | N.I. | N.I. |
| **R16, R17, R23, R24** | No | Yes | I | S | No | Yes* |
| **R21** | No | Yes | I | S | Yes | Yes* |
| **R26** | No | Yes | I | S | N.I. | Yes* |
| **R28, R29** | No | Yes | I | I | N.I. | Yes* |
| **R49** | No | Yes | I | I | N.I. | N.I. |
| **Y1, Y7, Y18** | No | No | N.I. | N.I. | N.I. | N.I. |
| **Y13** | No | Yes | +/- | S | No | Yes |
| **Y17** | No | Yes | +/- | S | No | Yes |
| **Z3, Z5, Z7, Z8, Z21, Z43, Z47** | Yes | N.I. | N.I. | N.I. | N.I. | N.I. |

^a^ScN2a cells were treated with the compounds for four days. Then they were evaluated by optical microscopy and the compounds that induced evident cell damage/loss were not further investigated (N.I.).

^b^The compounds that reduced PrP-Res levels in ScN2a cells by more than 50% at concentration ≤ 10 µM were classified as effective. The compounds that did not fit this rule were excluded from the next analyses.

^c^The pharmacokinetic (PK) properties of the compounds were evaluated *in silico* with ACD/Percepta Platform, Osiris and ChemSilico software. The compounds that showed oral bioavailability ≥70% and were predicted as non-mutagenic were classified as satisfactory (S). Compounds that not fit these criteria were considered insufficient (I).

^d^Physicochemical properties were predicted *in silico* with ACD/Percepta Platform, Osiris and ChemSilico software. Compounds were classified as sufficiently permeable in the CNS (S) or inactive (I) due to low penetration.

^e^Cytotoxicity to N2a cells was evaluated by MTT reduction assay. Compounds that caused cell death at concentrations ≤ 10 µM were considered cytotoxic.

^f^To verify the ability to interact directly with PrP, compounds were evaluated in an *in vitro* assay with PrP^109-149^ peptide; in a cell-free conversion assay (RT-QuIC); and also *in silico*, by molecular docking. * indicates compounds that were evaluated only by *in silico* methods.

N.I., not investigated
